# Supplementary material for: Glucocorticoid receptor triggers a reversible drug-tolerant dormancy state with acquired therapeutic vulnerabilities in lung cancer
Source: Nat Commun. 2021 Jul 16;12:4360. doi: 10.1038/s41467-021-24537-3 (PMC8285479; doi:10.1038/s41467-021-24537-3)
Supplement: Supplementary file 3 — Description of Additional Supplementary Files [file 41467_2021_24537_MOESM3_ESM.pdf]

Description of Additional Supplementary Files

File Name: Supplementary Data 1
Description: Drug screen data for H1944 cell line.

File Name: Supplementary Data 2
Description: RIME analysis results for p57 vs IgG performed in H2122 cell line.

File Name: Supplementary Data 3
Description: RIME analysis results for GR vs IgG performed in A549, H2122, H1944, H1975 and H460 cell lines.

File Name: Supplementary Data 4
Description: Comparative analysis of GR-RIME data in three GC-responsive (A549, H2122 and H1944) vs. two GC-resistant (H1975 and H460) cell lines.
