# Supplementary material for: Glucocorticoid receptor triggers a reversible drug-tolerant dormancy state with acquired therapeutic vulnerabilities in lung cancer
Source: Nat Commun. 2021 Jul 16;12:4360. doi: 10.1038/s41467-021-24537-3 (PMC8285479; doi:10.1038/s41467-021-24537-3)
Supplement: Supplementary file 8 — Reporting Summary [file 41467_2021_24537_MOESM8_ESM.pdf]

nature research

Corresponding author(s): Stefan Prekovic and Wilbert Zwart

Last updated by author(s): Feb 6, 2021

Reporting Summary

Nature Research wishes to improve the reproducibility of the work that we publish. This form provides structure for consistency and transparency in reporting. For further information on Nature Research policies, see our Editorial Policies and the Editorial Policy Checklist.

Statistics

For all statistical analyses, confirm that the following items are present in the figure legend, table legend, main text, or Methods section.

n/a	Confirmed	
☐	☒	The exact sample size (*n*) for each experimental group/condition, given as a discrete number and unit of measurement
☐	☒	A statement on whether measurements were taken from distinct samples or whether the same sample was measured repeatedly
☐	☒	The statistical test(s) used AND whether they are one- or two-sided<br>*Only common tests should be described solely by name; describe more complex techniques in the Methods section.*
☐	☒	A description of all covariates tested
☐	☒	A description of any assumptions or corrections, such as tests of normality and adjustment for multiple comparisons
☐	☒	A full description of the statistical parameters including central tendency (e.g. means) or other basic estimates (e.g. regression coefficient) AND variation (e.g. standard deviation) or associated estimates of uncertainty (e.g. confidence intervals)
☐	☒	For null hypothesis testing, the test statistic (e.g. *F*, *t*, *r*) with confidence intervals, effect sizes, degrees of freedom and *P* value noted<br>*Give P values as exact values whenever suitable.*
☒	☐	For Bayesian analysis, information on the choice of priors and Markov chain Monte Carlo settings
☒	☐	For hierarchical and complex designs, identification of the appropriate level for tests and full reporting of outcomes
☒	☐	Estimates of effect sizes (e.g. Cohen's *d*, Pearson's *r*), indicating how they were calculated

Our web collection on statistics for biologists contains articles on many of the points above.

Software and code

Policy information about availability of computer code

Data collection	RNA seq: Strand-specific libraries were generated with the TruSeq Stranded mRNA sample preparation kit (Illumina, Part # 15031047 Rev. E) and sequenced on a HiSeq2500 (65bp reads, single-end).<br>ChIP seq: Samples were sequenced using an Illumina Hiseq2500 genome analyser (65bp reads, single-end).<br>4C analysis: 4C libraries were sequenced on a MiSeq machine.<br>Seahorse assay: Cellular respiration was measured using a Seahorse XF24 Bioanalyzer (Seahorse Biosciences).<br>Flow cytometry data: data acquisition was performed on LSRFortessa SORP 2 (BD BioSciences) flow cytometer or Attune NxT Flow Cytometer (Thermo Fisher Scientific, USA) .<br>Mass spectrometry: LC-MS/MS analysis of the tryptic digests was performed on an Orbitrap Fusion Tribrid mass spectrometer equipped with a Proxeon nLC1000 system (Thermo Scientific) or peptide mixtures were analysed by nanoLC-MS/MS on an Q Exactive HF-X Hybrid Quadrupole-Orbitrap Mass Spectrometer equipped with an EASY-NLC 1200 system (Thermo Scientific).
Data analysis	For ChIP-seq data, raw Illumina sequencing data were aligned with BWA v0.5.20 to hg19. Following that the reads were filtered based on MAPQ quality ((samtools v1.8); quality ≥ 20) and duplicate reads were removed (Picard MarkDupes v2.18). Peak calling over input control was performed using MACS2 (v2.1.1) peak caller. MACS2 was run with the default parameters. Genome browser snapshots, heatmaps and density plots were generated using EaSeq (http://easeq.net). RNA sequencing data was mapped to exons using Tophat (v.2.1) and RNA-seq counts were normalised for library size using the DESeq2 (v1.22.2) package in R (v.3.5.0). All the plots were generated using ggplot2 (v3.3.2). For 4C analysis we used a customized pipeline (https://github.com/deWitLab/4C_mapping). Flow cytometry data has been analysed using FlowJo Software (FlowJo LLC, USA). Raw proteomics data were analysed by Proteome Discoverer (PD) (v. 2.3.0.523, Thermo Scientific) using standard settings. Phosphoproteome data were analysed by MaxQuant (v. 1.6.1.0) using standard settings.<br><br>BWA 0.7.17 - http://bio-bwa.sourceforge.net/) for mapping;<br>MACS2 2.1.2 - http://liulab.dfci.harvard.edu/MACS/ for peak calling;<br>DEseq2 1.28.0 - https://bioconductor.org/packages/release/bioc/html/DESeq2.html for differential peak analysis.

4C_mapping tool - https://github.com/deWitLab/4C_mapping for 4C mapping.
EaSeq - http://easeq.net

For manuscripts utilizing custom algorithms or software that are central to the research but not yet described in published literature, software must be made available to editors and reviewers. We strongly encourage code deposition in a community repository (e.g. GitHub). See the Nature Research guidelines for submitting code & software for further information.

Data

Policy information about availability of data

All manuscripts must include a data availability statement. This statement should provide the following information, where applicable:

- Accession codes, unique identifiers, or web links for publicly available datasets
- A list of figures that have associated raw data
- A description of any restrictions on data availability

All genomic and mass spectrometry data generated in this study have been deposited in the Gene Expression Omnibus (GEO) and Proteomics Identification (PRIDE) databases, under accession numbers GSE159546 (https://www.ncbi.nlm.nih.gov/geo/query/acc.cgi?acc=GSE159546) and PXD021924 (https://www.ebi.ac.uk/pride/archive/projects/PXD021924), respectively. Public datasets used in this study are available from GEO or ENCODE, archived under the following codes: U01HG007900 (https://www.encodeproject.org/awards/U01HG007900; A549 time-series experiments), GSE24397 (https://www.ncbi.nlm.nih.gov/geo/query/acc.cgi?acc=GSE24397; HeLa ChIP sequencing experiments), and GSE49591 (https://www.ncbi.nlm.nih.gov/geo/query/acc.cgi?acc=GSE49591; HeLa microarray experiments). The remaining data are available within the Article, Supplementary Information or available from the authors upon.

Field-specific reporting

Please select the one below that is the best fit for your research. If you are not sure, read the appropriate sections before making your selection.

☒ Life sciences ☐ Behavioural & social sciences ☐ Ecological, evolutionary & environmental sciences

For a reference copy of the document with all sections, see nature.com/documents/nr-reporting-summary-flat.pdf

Life sciences study design

All studies must disclose on these points even when the disclosure is negative.

Sample size	Sample size was based on previous experience with the mouse models (Handle, F. et al 2019) or otherwise determined using G*Power software version 3.1.<br>For all the experiments in the manuscript more than 2 - 3 independent replicates were generated.<br>Number of cell lines analyses was based on responsiveness (or not) to GC treatment.<br>Number of replicates for next generation sequencing related experiments was based on guidelines defined by the ENCODE consortium.
Data exclusions	No data was excluded from the analysis.
Replication	In vitro experiments were repeated in at least 2 - 3 independent experiments and showed comparable results between experiments.
Randomization	For intervention studies, mice were randomly distributed over the treatment arms when tumours reached the size indicated in the figures. The first animal was assigned randomly in the control or treatment groups, after which each subsequent animal was placed in the next group.
Blinding	Tumor measurements analyses were performed in a blinded fashion.

Reporting for specific materials, systems and methods

We require information from authors about some types of materials, experimental systems and methods used in many studies. Here, indicate whether each material, system or method listed is relevant to your study. If you are not sure if a list item applies to your research, read the appropriate section before selecting a response.

Materials & experimental systems

n/a	Involved in the study	
☐	☒	Antibodies
☐	☒	Eukaryotic cell lines
☒	☐	Palaeontology and archaeology
☐	☒	Animals and other organisms
☒	☐	Human research participants
☒	☐	Clinical data
☒	☐	Dual use research of concern

Methods

n/a	Involved in the study	
☐	☒	ChIP-seq
☒	☐	Flow cytometry
☒	☐	MRI-based neuroimaging

Antibodies

Antibodies used	- anti-GR (12041, Cell Signalling Technology)<br>- anti-p57 (sc-56341, Santa Cruz Biotechnology)<br>- anti-rabbit IgG (sc-2027, Santa Cruz Biotechnology)<br>- anti-mouse IgG (sc-2025, Santa Cruz Biotechnology)<br>- anti-actin (MAB1501R, Merck, 1:10,000)<br>- anti-AR (#06680, Merck Millipore, 1:1000)<br>- anti-ER (MA5 14104, Thermo Fisher Scientific)<br>- anti-PR (sc-539, Santa Cruz Biotechnology)<br>- anti-PARP (9542, Cell Signalling Technology)<br>- anti-p53 (sc-126, Santa Cruz Biotechnology)<br>- anti-Hsp90 (sc-13119, Santa Cruz Biotechnology)<br>- anti-Ki67 (ab155580, Abcam)<br>- anti- phospho-IGF-1R (Y1161, ab39398, Abcam)<br>- anti-p21 (sc-6246, Santa Cruz)<br>- anti-Rb (ab181616, Abcam)<br>- anti-phospho-Rb (Ser780, #9307, Cell Signalling)<br>- anti-cleaved caspase-3 (#9661, Cell Signalling)<br>- Goat anti-Rabbit IgG (H+L) Cross-Adsorbed Secondary Antibody, Alexa Fluor 647 (A21244, Thermo Fisher Scientific)<br>- Goat anti-Mouse IgG (H+L) Cross-Adsorbed Secondary Antibody, Alexa Fluor 488 (A11001, Thermo Fisher Scientific)
Validation	GR and p57 antibodies were validated in various experiments using CRISPR-knockout models for GR of p57, respectively. For this we used immuno-floursecence (GR and p57) and ChIP (GR) experiments.<br>Antibodies used for IHC were validated for mouse by the Pathology facilities at the NKI.<br>All the other anybodies were validated using western blots, and mass spec experiments using isotype controls when necessary.

Eukaryotic cell lines

Policy information about cell lines

Cell line source(s)	A549, H2122, H1944, H1975, and H460 cells were obtained from Rene Bernard's lab (Netherlands Cancer Institute, Netherlands). These cells were originally obtained from American Type Culture Collection (ATCC). HEK293T and HeLa cells were obtained from American Type Culture Collection (ATCC). The human mesothelioma cell lines M28 and VAMT were provided by Courtney Broaddus (University of California, USA), while the NCI-H2795 (H2795) was obtained from Ultan McDermott (Sanger Institute, UK). The primary human mesothelioma cell lines PV130913, PV041214, , PV020318, PV150318, PV240418, PV180518, and PV250518 were generated by Laurel Schunselaar (Netherlands Cancer Institute, Netherlands).
Authentication	All the cell lines were authenticated using STR profiling.
Mycoplasma contamination	All the cell lines were tested negative for Mycoplasma contamination.
Commonly misidentified lines<br>(See ICLAC register)	No commonly misidentified cell lines were used in the study.

Animals and other organisms

Policy information about studies involving animals; ARRIVE guidelines recommended for reporting animal research

Laboratory animals	Male NOD-scid-g (NSG) mice, ±7 weeks old.
Wild animals	No wild animals were used in the study.
Field-collected samples	No field-collected samples were used in the study.
Ethics oversight	The NKI Animal Experiments Committee approved all in vivo experiments (project number 9139 and 9907).

Note that full information on the approval of the study protocol must also be provided in the manuscript.

ChIP-seq

Data deposition

☒ Confirm that both raw and final processed data have been deposited in a public database such as GEO.

☒ Confirm that you have deposited or provided access to graph files (e.g. BED files) for the called peaks.

Data access links<br>*May remain private before publication.*	All the data has been deposited to GEO and can be found as a part of the following series.<br>https://www.ncbi.nlm.nih.gov/geo/query/acc.cgi?acc=GSE159546

Files in database submission	A549-DCC-peaks-rep1.narrowPeak<br>4766_3_wz2428_AGATCGCA_S32.bam<br>A549-HC-peaks-rep1.narrowPeak<br>4766_4_wz2429_AGCAGGAA_S33.bam<br>H2122-DCC-peaks-rep1.narrowPeak<br>4766_19_wz2444_CGACTGGA_S48.bam<br>H2122-HC-peaks-rep1.narrowPeak<br>4766_20_wz2445_CGCATACA_S49.bam<br>H460-DCC-peaks-rep1.narrowPeak<br>4766_11_wz2436_CACTTCGA_S40.bam<br>H460-HC-peaks-rep1.narrowPeak<br>4766_12_wz2437_CAGCGTTA_S41.bam<br>H1944-DCC-peaks-rep1.narrowPeak<br>5041_2_wz2932_TAGGATGA_S62.bam<br>H1944-HC-peaks-rep1.narrowPeak<br>5041_3_wz2933_TCTTCACA_S63.bam<br>H1975-DCC-peaks-rep1.narrowPeak<br>5041_5_wz2935_TTCACGCA_S65.bam<br>H1975-HC-peaks-rep1.narrowPeak<br>5041_6_wz2936_AAGGACAC_S66.bam<br>A549-DCC-peaks-rep2.narrowPeak<br>4798_17_wz2490_ATGCCTAA_S45.bam<br>A549-HC-peaks-rep2.narrowPeak<br>4798_18_wz2491_AGTGGTCA_S46.bam<br>H2122-DCC-peaks-rep2.narrowPeak<br>4798_13_wz2486_GATGAATC_S41.bam<br>H2122-HC-peaks-rep2.narrowPeak<br>4798_14_wz2487_GCCAAGAC_S42.bam<br>H460-DCC-peaks-rep2.narrowPeak<br>4798_11_wz2484_GACAGTGC_S39.bam<br>H460-HC-peaks-rep2.narrowPeak<br>4798_12_wz2485_GAGTTAGC_S40.bam
Genome browser session (e.g. UCSC)	https://genome.ucsc.edu/s/StefanPrekovic/GR_ChIP_LungCancer

Methodology

Replicates	GR-ChIP sequencing was performed in five different lung cancer cell lines. For A549, H2122, and H460 two biological replicates were used, and for H1944 and H1975 one biological replicate was performed.
Sequencing depth	On average each experiment had 28047548 single-end reads of 65 bps, out of which 93% were mapped with the highest and most occurring quality of 37.
Antibodies	NR3C1 antibody (D6H2L (lot #3), Cell Signalling Technology)
Peak calling parameters	Default settings of MACS2 were used as follows: callpeak -t sample.bam -c control.bam -f BAM -g hs -n test -B -q 0.01
Data quality	We evaluated multiple quality control criteria based on alignment information and peak quality: (i) sequence quality score; (ii) uniquely mappable reads (reads that can only map to one location in the genome); (iii) uniquely mappable locations (locations that can only be mapped by at least one read); (iv) number of total peaks; (v) high-confidence peaks (the number of peaks that are tenfold enriched over background); (vi) percentage overlap with known DHS sites derived from the ENCODE Project (the minimum required to meet the threshold was 80%); and (viii) peak conservation (a measure of sequence similarity across species based on the hypothesis that conserved sequences are more likely to be functional). Typically, if a sample fails one of these criteria, it will fail many (locations with low mappability will likely have low peak numbers, many of which will likely be in high-mappability regions, etc.).
Software	All ChIP seq data was analysed using Easeq software (https://easeq.net).
