# Supplementary material for: Glucocorticoid receptor triggers a reversible drug-tolerant dormancy state with acquired therapeutic vulnerabilities in lung cancer
Source: Nat Commun. 2021 Jul 16;12:4360. doi: 10.1038/s41467-021-24537-3 (PMC8285479; doi:10.1038/s41467-021-24537-3)
Supplement: Supplementary file 1 — Supplementary Information [file 41467_2021_24537_MOESM1_ESM.pdf]

Supplementary information

Glucocorticoid receptor triggers a reversible drug-tolerant dormancy state with acquired therapeutic vulnerabilities in lung cancer

Stefan Prekovic[1,#,*], Karianne Schuurman[1,#], Isabel Mayayo-Peralta[1,$], Anna G. Manjón[2,$], Mark Buijs[1], Selçuk Yavuz[1], Max D. Wellenstein[3], Alejandro Barrera[4], Kim Monkhorst[5], Anne Huber[1,6], Ben Morris[7], Cor Lieftink[7], Theofilos Chalkiadakis[1], Ferhat Alkan[1], Joana Silva[1], Balázs Győrffy[8,9], Liesbeth Hoekman[10], Bram van den Broek[11], Hans Teunissen[12], Donna O. Debets[13], Tesa Severson[1], Jos Jonkers[14], Timothy Reddy[4], Karin E. de Visser[3], William Faller[1], Roderick Beijersbergen[7], Maarten Altelaar[10,13], Elzo de Wit[11], Rene Medema[2], and Wilbert Zwart[1,15,*]

[1]Division of Oncogenomics, Oncode Institute, The Netherlands Cancer Institute, Amsterdam, The Netherlands
[2]Division of Cell Biology, Oncode Institute, The Netherlands Cancer Institute, Amsterdam, The Netherlands
[3]Division of Tumour Biology and Immunology, Oncode Institute, The Netherlands Cancer Institute, Amsterdam, The Netherlands
[4]Department of Biostatistics & Bioinformatics, and Centre for Genomic & Computational Biology, Duke University Medical Centre, Durham, United States
[5]Department of Pathology, The Netherlands Cancer Institute, Amsterdam, The Netherlands
[6]Current address: Olivia Newton-John Cancer Research Institute and School of Cancer Medicine, La Trobe University, Melbourne, Australia
[7]Division of Molecular Carcinogenesis and Robotics and Screening Centre, Netherlands Cancer Institute, Amsterdam, The Netherlands
[8]Semmelweis University Dept. of Bioinformatics and 2nd Dept of Pediatrics, Budapest, Hungary
[9]TTK Cancer Biomarker Research Group, Institute of Enzymology, Budapest, Hungary
[10]Mass spectrometry/Proteomics Facility, The Netherlands Cancer Institute, Amsterdam, The Netherlands
[11]Division of Cell Biology and BioImaging Facility, The Netherlands Cancer Institute, Amsterdam, The Netherlands
[12]Division of Gene Regulation, Oncode Institute, The Netherlands Cancer Institute, Amsterdam, The Netherlands
[13]Biomolecular Mass Spectrometry and Proteomics, Bijvoet Center for Biomolecular Research and Utrecht Institute for Pharmaceutical Sciences, Utrecht University, Utrecht, The Netherlands
[14]Division of Molecular Pathology, Oncode Institute, The Netherlands Cancer Institute, Amsterdam, The Netherlands
[15]Laboratory of Chemical Biology and Institute for Complex Molecular Systems, Department of Biomedical Engineering, Eindhoven University of Technology, Eindhoven, The Netherlands

[#]Authors contributed equally [$]Shared second author *Corresponding authors

Stefan Prekovic, Division of Oncogenomics, Oncode Institute, Netherlands Cancer Institute, 1066CX Amsterdam, The Netherlands. Phone: +31 205122023. e-mail: s.prekovic@nki.nl
Wilbert Zwart, Division of Oncogenomics, Oncode Institute, Netherlands Cancer Institute, 1066CX Amsterdam, The Netherlands. Phone: +31 205122101. e-mail: w.zwart@nki.nl

Supplementary Table 1. Specific treatment information for each figure in the manuscript. The type of glucocorticoid, concentration and duration of treatment are indicated.

Figure	Panel	Glucocorticoid	Concentration	Time
1	a	Hydrocortisone	2,75 uM	8 hrs
1	b	Hydrocortisone	2,75 uM	6 days
1	c	Hydrocortisone	2,75 uM	48 hrs
1	d	Hydrocortisone	2,75 uM	48 hrs
1	e	Hydrocortisone	2,75 uM	48 hrs
1	f	Hydrocortisone	2,75 uM	48 hrs
1	g	Hydrocortisone	2,75 uM	48 hrs
1	h	Hydrocortisone	2,75 uM	48 hrs
1	i	Hydrocortisone	2,75 uM	48+ hrs
1	j	Hydrocortisone	2,75 uM	48+ hrs
2	a	Hydrocortisone	2,75 uM	6 days (co-treated) / 8 days (pre-treated)
2	b	Hydrocortisone	2,75 uM	6 days (co-treated) / 8 days (pre-treated)
2	c	NA	NA	NA
2	d	Hydrocortisone	2,75 uM	48 hrs
2	e	Dexamethasone	4 mg/kg	15 days
2	f	Dexamethasone	4 mg/kg	15 days
3	a	Hydrocortisone	2,75 uM	8 hrs
3	b	Hydrocortisone	2,75 uM	8 hrs
3	c	Hydrocortisone	2,75 uM	8 hrs
3	d	Hydrocortisone	2,75 uM	8 hrs
3	e	Dexamethasone	100 nM	1-12 hrs
3	f	Dexamethasone	100 nM	1-12 hrs
3	g	Hydrocortisone	2,75 uM	8 hrs
3	h	Hydrocortisone	2,75 uM	60 hrs
3	i	Hydrocortisone	2,75 uM	8 hrs
3	j	Hydrocortisone	2,75 uM	8 hrs
3	k	Hydrocortisone	2,75 uM	8 hrs
4	a	Hydrocortisone	2,75 uM	2 hrs for ChIP / 8 hrs RNA seq
4	b	Dexamethasone	100 nM	2 hrs
4	c	Hydrocortisone	2,75 uM	2 hrs
4	d	Hydrocortisone	2,75 uM	2 hrs
4	e	Hydrocortisone	2,75 uM	24 hrs
4	f	Hydrocortisone	2,75 uM	5 days
5	a	Hydrocortisone	2,75 uM	8 hrs
5	b	Hydrocortisone	2,75 uM	2 hrs
5	c	Hydrocortisone	2,75 uM	8 hrs
5	d	Hydrocortisone	2,75 uM	2 hrs
5	e	Hydrocortisone	2,75 uM	2 hrs
5	f	Hydrocortisone	2,75 uM	2 hrs
5	g	NA	NA	NA
5	h	Hydrocortisone	2,75 uM	8 hrs
6	a	NA	NA	NA
6	b	NA	NA	NA
6	c	NA	NA	NA
6	d	NA	NA	NA
6	e	NA	NA	NA
7	NA	NA	NA	NA

Figure	Panel	Glucocorticoid	Concentration	Time
S1	a	NA	NA	NA
S1	b	Hydrocortisone	2,75 uM	2 days
S1	c	Hydrocortisone	2,75 uM	24 hrs
S1	d	Hydrocortisone	2,75 uM	48 hrs
S1	e	Hydrocortisone	2,75 uM	8 hrs for RNA seq / 48 hrs for proteomics
S1	f	Hydrocortisone	2,75 uM	5+ days
S1	g	Hydrocortisone	2,75 uM	48+ hrs
S1	h	NA	NA	NA
S1	i	NA	NA	NA
S1	j	NA	NA	NA
S1	k	NA	NA	NA
S1	l	NA	NA	NA
S1	m	NA	NA	NA
S2	a (top)	Hydrocortisone	2,75 uM	8 hrs
S2	a (bottom)	Hydrocortisone and Dexamethasone	Various	5 days
S2	b	Hydrocortisone	2,75 uM	48 hrs
S2	c	Hydrocortisone	2,75 uM	6 days
S2	d	NA	NA	NA
S2	e	NA	NA	NA
S2	f	Hydrocortisone and Dexamethasone	Various	5 days
S3	a	NA	NA	NA
S3	b	Hydrocortisone	2,75 uM	6 days (co-treated) / 8 days (pre-treated)
S3	c	Hydrocortisone	2,75 uM	6 days (co-treated) / 8 days (pre-treated)
S3	d	Hydrocortisone	2,75 uM	8 days
S3	e	NA	NA	NA
S3	f	Dexamethasone	4 mg/kg	15 days
S3	g	Dexamethasone	4 mg/kg	15 days
S3	h	Dexamethasone	4 mg/kg	15 days
S3	i	Dexamethasone	4 mg/kg	15 days
S4	a	Dexamethasone	4 mg/kg	25 days
S4	b	Dexamethasone	4 mg/kg	25 days
S4	c	Dexamethasone	100 nM	1-6 hrs
S4	d	NA	NA	NA
S4	e	NA	NA	NA
S4	f	Dexamethasone	100 nM	1-12 hrs
S4	g	Dexamethasone	100 nM	1-12 hrs
S5	a	Hydrocortisone	2,75 uM	8 hrs
S5	b	Hydrocortisone	2,75 uM	24 hrs
S5	c	Hydrocortisone	2,75 uM	8 hrs
S5	d	Hydrocortisone	2,75 uM	48 hrs
S5	e	NA	NA	NA
S5	f	NA	NA	NA
S5	g	Hydrocortisone	2,75 uM	8 hrs
S5	h	Hydrocortisone	2,75 uM	8 hrs
S5	i	Hydrocortisone	2,75 uM	8 hrs
S6	a	Hydrocortisone	2,75 uM	8 hrs
S6	b	Hydrocortisone	2,75 uM	2 hrs
S7	a	Hydrocortisone	Various	5 days
S7	b	Dexamethasone	100 nM	3 hrs
S7	c	Hydrocortisone	2,75 uM	8 hrs
S7	d	NA	NA	NA

Supplementary Table 2. Primer sequences used in the study. The table includes the sequences of primers used for RT-qPCR and 4C experiments.

Target	Sequence	Application
ARID1a_FW	ACCTCTATCGCCTCTATGTGTCTGT	RT-qPCR
ARID1a_RV	CTGGCAGCACTGCTTGATGT	RT-qPCR
ARID2_FW	GCCTGGAGATCCAATGAGAA	RT-qPCR
ARID2_RV	TCCTCCATGTTCAGTTGCTG	RT-qPCR
SMARCA2_FW	TCCGAGGCAAAATCAGTCAAG	RT-qPCR
SMARCA2_RV	TTCCTCGATTTGGCCTTTTCT	RT-qPCR
SMARCB1_FW	GCCTTCACCTGGAACATGAA	RT-qPCR
SMARCB1_RV	AACGTCAGCGGGTTCAAAT	RT-qPCR
SMARCC2_FW	TCTTCAACGGCAAGAACAAG	RT-qPCR
SMARCC2_RV	TACTCTTGGGGGTTCAGTCG	RT-qPCR
SMARCD2_FW	GATCCATTCCGAAAACGCCTG	RT-qPCR
SMARCD2_RV	TGAGGTAGAACCTTATCTGCCA	RT-qPCR
SMARCD3_FW	AACCAGCAGGAGATCAGTGC	RT-qPCR
SMARCD3_RV	GCCGGCTACATCTGTCATCA	RT-qPCR
SMARCE1_FW	AACAACTACAGGCTGGGAGG	RT-qPCR
SMARCE1_RV	CGGCTTATCTGGTGGCTTT	RT-qPCR
CDKN1C_FW	GCGGCGATCAAGAAGCTG	RT-qPCR
CDKN1C_RV	GCTTGGCGAAGAAATCGGAGA	RT-qPCR
GAPDH_FW	ATTTGGCTACAGCAACAGGG	RT-qPCR
GAPDH_RV	TCAAGGGGTCTACATGGCA	RT-qPCR
UBC FW	ATTTGGGTCGCGGTTCTTG	RT-qPCR
UBC RV	TGCCTTGACATTCTCGATGGT	RT-qPCR
Beta-Actin FW	CCTGGCACCCAGCACAAT	RT-qPCR
Beta-Actin RV	GGGCCGGACTCGTCATACT	RT-qPCR
chr11:2800588-2801154_FW	ATTTTCCGGAGTCCCTGATC	4C CDKN1C enhancer
chr11:2800588-2801154_RV	ATGTCTAGAAACTCCTGGCC	4C CDKN1C enhancer
chr11:2903059-2904509_FW	ACTGTTTGTGGTGACCGATC	4C CDKN1C promoter
chr11:2903059-2904509_RV	ATTTTTCGGCCTCTTTATTT	4C CDKN1C promoter

Supplementary Figure 1.

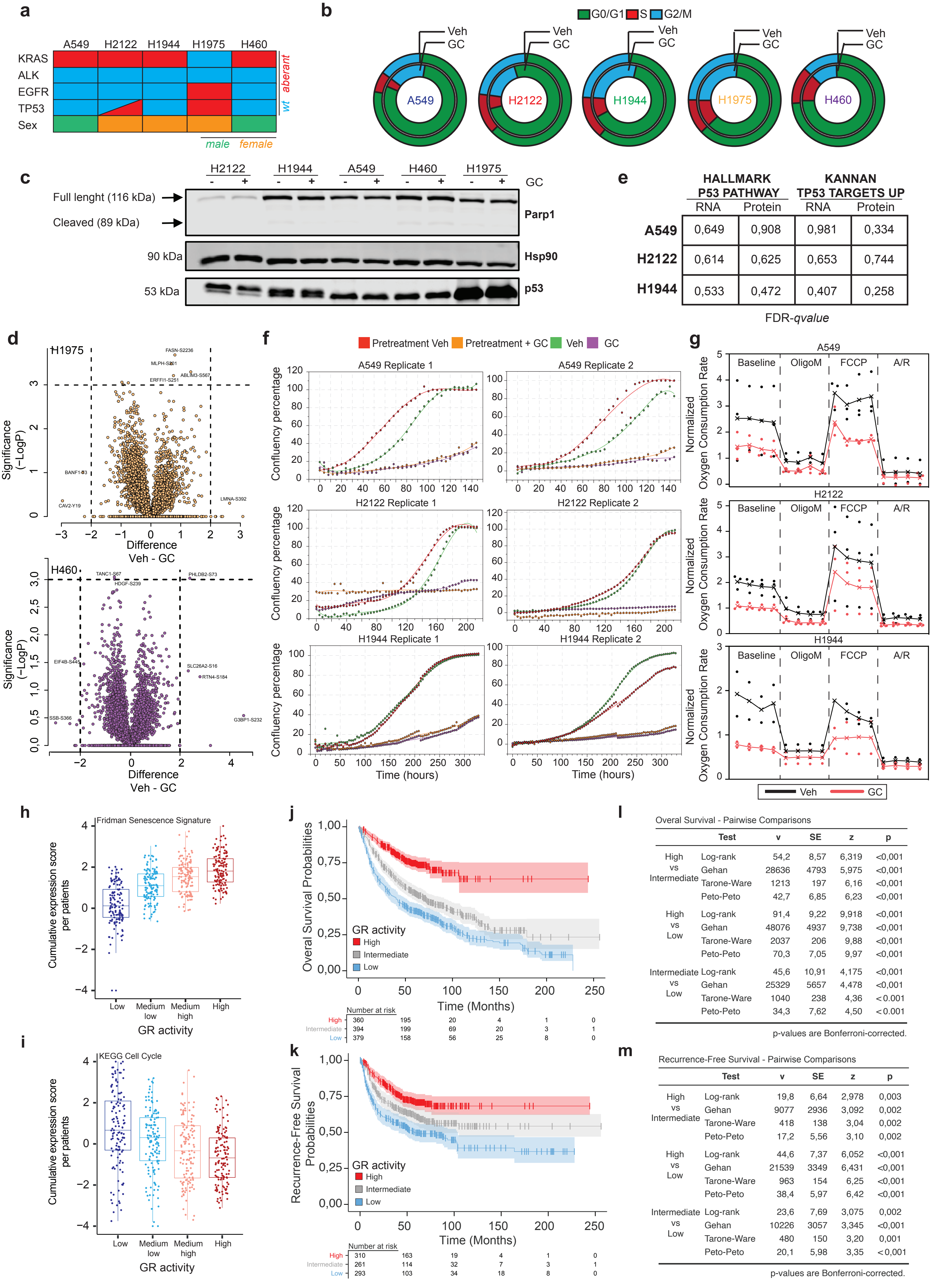

e

	HALLMARK P53 PATHWAY		KANNAN TP53 TARGETS UP	
	RNA	Protein	RNA	Protein
**A549**	0,649	0,908	0,981	0,334
**H2122**	0,614	0,625	0,653	0,744
**H1944**	0,533	0,472	0,407	0,258

FDR-*qvalue*

l

Overal Survival - Pairwise Comparisons

	Test	v	SE	z	p
High vs Intermediate	Log-rank	54,2	8,57	6,319	<0,001
	Gehan	28636	4793	5,975	<0,001
	Tarone-Ware	1213	197	6,16	<0,001
	Peto-Peto	42,7	6,85	6,23	<0,001
High vs Low	Log-rank	91,4	9,22	9,918	<0,001
	Gehan	48076	4937	9,738	<0,001
	Tarone-Ware	2037	206	9,88	<0,001
	Peto-Peto	70,3	7,05	9,97	<0,001
Intermediate vs Low	Log-rank	45,6	10,91	4,175	<0,001
	Gehan	25329	5657	4,478	<0,001
	Tarone-Ware	1040	238	4,36	< 0.001
	Peto-Peto	34,3	7,62	4,50	< 0.001

p-values are Bonferroni-corrected.

m

Recurrence-Free Survival - Pairwise Comparisons

	Test	v	SE	z	p
High vs Intermediate	Log-rank	19,8	6,64	2,978	0,003
	Gehan	9077	2936	3,092	0,002
	Tarone-Ware	418	138	3,04	0,002
	Peto-Peto	17,2	5,56	3,10	0,002
High vs Low	Log-rank	44,6	7,37	6,052	<0,001
	Gehan	21539	3349	6,431	<0,001
	Tarone-Ware	963	154	6,25	<0,001
	Peto-Peto	38,4	5,97	6,42	<0,001
Intermediate vs Low	Log-rank	23,6	7,69	3,075	0,002
	Gehan	10226	3057	3,345	<0,001
	Tarone-Ware	480	150	3,20	0,001
	Peto-Peto	20,1	5,98	3,35	< 0,001

p-values are Bonferroni-corrected.

Supplementary Figure 1. Characterisation of glucocorticoid-induced cancer cell dormancy in lung cancer models and patient samples.

a, Characteristics of cell lines used in the study, depicting mutation status of key lung cancer drivers (wt=blue or aberrant=red) and sex of origin (male=green or female=orange). **b,** Cell cycle distribution by phase (G0/G1=green, S=red, and G2/M=blue) of untreated (Veh) or glucocorticoid treated (GC) cells. **c,** Representative western blot showing (cleaved) PARP as well as p53 expression, with Hsp90 as loading control (n=2). **d,** Volcano plots depicting differential protein phosphorylation in H1975 (top) and H460 (bottom) for comparison of vehicle (Veh) and GC treatment (n=3). p-values were determined by two-sided t-test. **e,** GSEA FDR-q-values for two p53-related gene-sets (M5939 and M16229) of transcriptomic and proteomic datasets for comparison of vehicle and GC treatment (n=2 for RNA sequencing and n=3 for mass spectrometry experiments). FDR-q-values were determined by GSEA software. **f,** Confluency percentage of cells following a five-day pre-treatment with vehicle (continued on Veh=green and GC=purple) or GCs (continued on Veh=red and GC=orange). Mean values of technical replicates of two independent biological replicates are depicted side-by-side. **g,** Per DNA content normalized oxygen consumption rate. For each condition four timepoints were measured. OligoM = Oligomycin; FCCP = arbonyl cyanide-4-(trifluoromethoxy)phenylhydrazone; A/R = Antimycin A/Rotenone. Vehicle (Veh=black) and glucocorticoid (GC=red) treatment was used. Cross represents mean value (A549 n=3, H2122 n=3, H1944 n=2). **h,** Expression score for Fridman Senescence Signature (M9143) in human lung adenocarcinoma samples (n=515) grouped based on GR activity (25% split). The central mark indicates the median, and the bottom and top edges of the box indicate the 25th and 75th percentiles, respectively. The maximum whisker lengths are specified as 1,5 times the interquartile range. **i,** Expression score for KEGG Cell Cycle gene-set (hsa04110) in human lung adenocarcinoma sample (n=515) grouped based on GR activity (25% split). The central mark indicates the median, and the bottom and top edges of the box indicate the 25th and 75th percentiles, respectively. The maximum whisker lengths are specified as 1,5 times the interquartile range. **j,** Overall survival probabilities of lung cancer patients (n=1529) grouped by transcriptomics-based GR activity signature (high=red, intermediate=grey, and low=blue). Overall survival

probabilities in months is plotted for each group, censored patients are shown as vertical tick marks and the Confidence Interval is shown as the shaded area around the respective line. **k,** Recurrence-free survival probabilities of lung cancer patients (n=1529) grouped by transcriptomics-based GR activity signature (high=red, intermediate=grey, and low=blue). Recurrence-free survival probabilities in months is plotted for each group, censored patients are shown as vertical tick marks and the Confidence Interval is shown as the shaded area around the respective line. **l,** Pairwise comparison of overall survival data depicted in panel **j**. p-values (two-sided) are Bonferroni-corrected. **m,** Pairwise comparison of recurrence-free survival data depicted in panel **k**. p-values (two-sided) are Bonferroni-corrected. Source data are provided as a Source Data file.

Supplementary Figure 2.

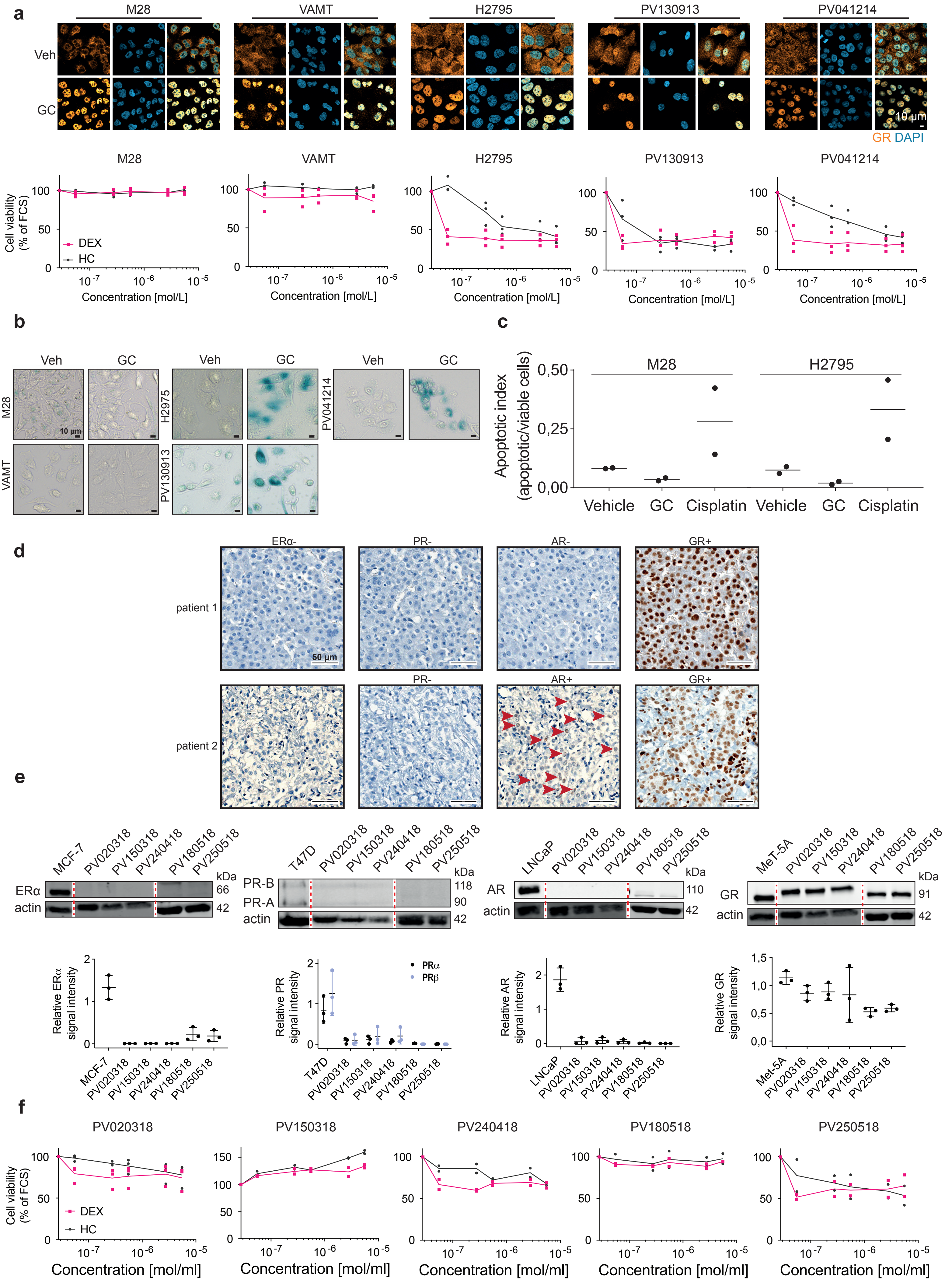

Supplementary Figure 2. Features of glucocorticoid response of mesothelioma models.

A (top), Representative immunofluorescence images showing expression and localisation of GR (orange), treated with glucocorticoids (GC) or control (Veh), using DAPI as nuclear staining (light blue) in mesothelioma cell lines (n=3). Scale bar, 10 μm. **a (bottom),** Cell viability assays of mesothelioma cell lines treated with increasing concentrations of glucocorticoids, hydrocortisone (HC=black) or dexamethasone (DEX=pink). Mean values of independent biological replicates depicted as a line (n=3). **b**, Representative images of senescence-associated β-galactosidase stained mesothelioma cell lines, untreated (Veh) or glucocorticoid treated (GC) (n=3). Scale bar, 10 μm. **c,** Ratio of apoptotic to viable cells in two mesothelioma cell lines untreated (Veh), or treated with glucocorticoids (GC) or cisplatin (n=2). **d,** Representative estrogen receptor (ERα), progesterone receptor (PR), androgen receptor (AR) and GR immunohistochemical staining of biopsies from 2 patients with mesothelioma. No expression of ERα and PR was observed. AR expression was observed in the second biopsy (brown staining, arrows) but not in the first biopsy. For GR, intense nuclear staining was observed. Original magnification 40x. Scale bar, 50 μm. **e,** Immunoblot analyses of ERα, PR-A/B, AR, GR in five primary MPM cells obtained from pleural fluid of patients (PV020318, PV150318, PV240418, PV180518, PV250518). As a control, we used ERα-positive MCF-7 human breast cancer cells, PR-A/B-positive T47D human breast cancer cells, AR-positive LNCaP human prostate cancer cells, and GR-positive MeT 5A human mesothelial cells. Actin is included as a loading control. For each hormone receptor, one representative immunoblot image out of three independent replicates taken from different passages of the same primary cell culture is shown. Graphs show relative hormone receptor signal intensity (normalised to loading control). Mean values ±SD of independent biological replicates is depicted (n=3). **f,** Dose-response curves depicting the cell viability (in percentage, normalised to untreated cells) of primary mesothelioma cells obtained from pleural fluid of patients over different concentrations of the GR agonists hydrocortisone (HC=black) or dexamethasone (DEX=pink). Mean values of independent biological replicates depicted as a line (n=2; except PV020318 n=3). Source data are provided as a Source Data file.

Supplementary Figure 3.

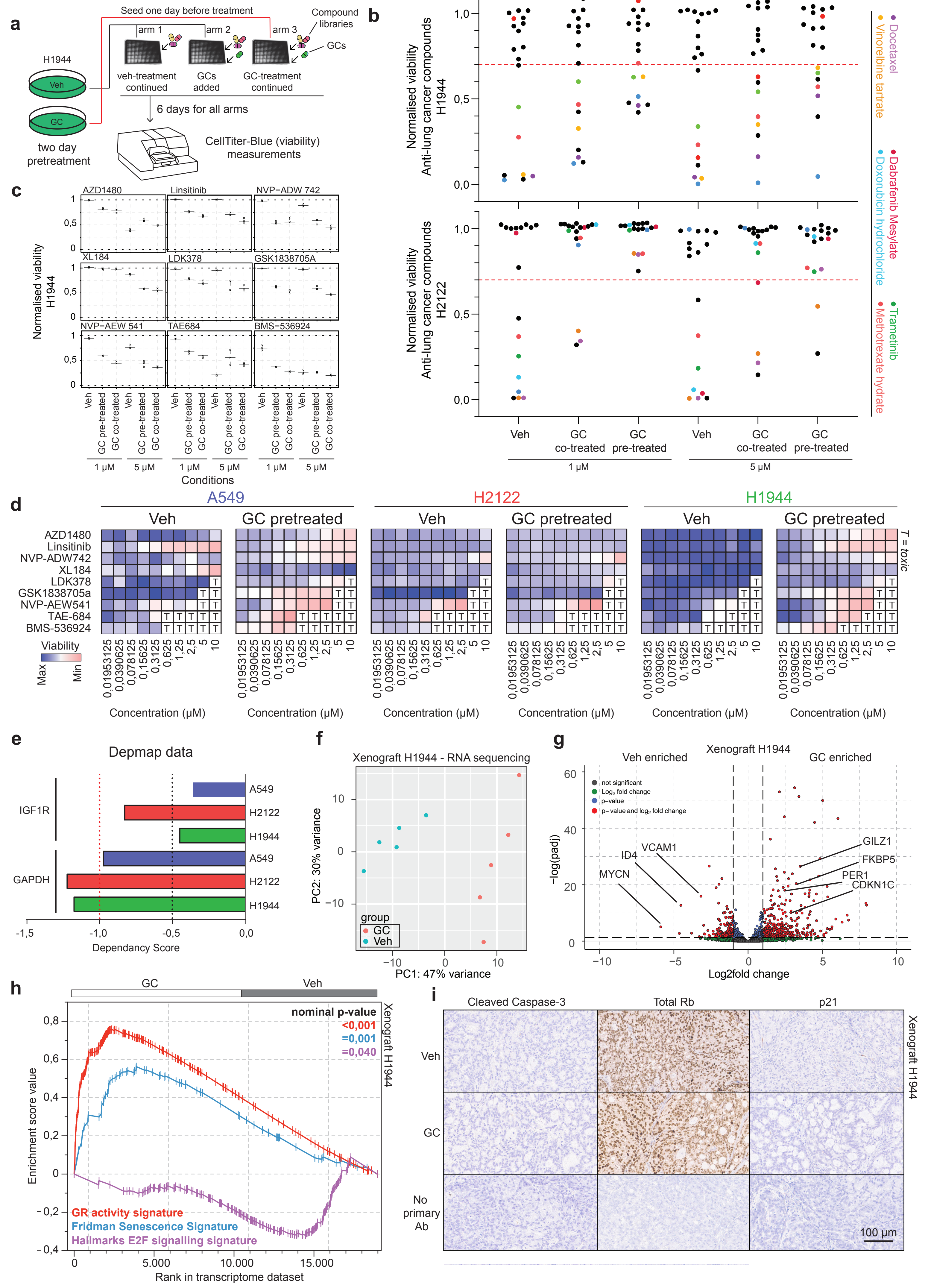

Supplementary Figure 3. Glucocorticoids alter drug sensitivity *in vitro* and evoke a transcriptional response in xenograft models.

a, Schematic representation of the compound screen. The cells were pre-treated with either vehicle or GCs for two days. Subsequently, cells were seeded in 384-well plates continuing respective treatments. After 24 hours, the vehicle pre-treated cells were either continued on vehicle treatment (arm1: vehicle) or stimulated with GCs (arm2: GC co-treatment), while the GC-pre-treated (arm3: GC pre-treatment) ones were kept in the same condition. On the same day, the compound library containing 2277 unique drugs was added to each of the arms in two concentrations (1 µM or 5 µM). After six days, the cell viability in all arms was measured with CellTiter-Blue assay using Envision Multilabel Plate Reader. **b,** Scatter plot depicting normalised viability of H1944 (top) and H2122 (bottom) cells vehicle-treated (Veh), GC co-treated and GC pre-treated in combination with 18 compounds approved for use in treatment of lung cancer (n=3). **c,** Plot depicting normalised viability for H1944 cell line treated with compounds found to have a significant degree of enhancement when combined with GCs, across all the arms of the screen. Mean values are shown as a line (n=3). **d,** Heatmaps depicting cell viability validation experiments for compound screen hits in A549, H2122, and H1944 cell lines (n=2). **e,** DepMap dependency scores for *IGF-1R* and *GAPDH* for A549, H2122, and H1944 cell lines. **f,** Principal component analysis of RNA sequencing data from H1944 xenograft tumour samples (n=5) from animals treated with either vehicle (Veh=blue) or glucocorticoids (GC=red). **g,** Volcano plot depicting transcriptional differences between vehicle and GC-treated H1944 xenograft tumours (n=5). Adjusted p-values (padj) were determined by DESeq2 (Wald test p-values corrected for multiple testing using Benjamini and Hochberg method). **h,** GR activity signature (red), Fridman Senescence signature (M9143; blue), and E2F target gene-set (M5925; purple) Gene-set enrichment analysis (GSEA) profiles for RNA sequencing experiments of H1944 xenograft tumours (n=5). Nominal p-values were determined by GSEA software. **i,** Representative images showing immunohistochemical stainings of H1944 xenograft cancer samples (n=4) obtained after treatment of NOD-SCID-γ mice with either vehicle (Veh) or glucocorticoids (GC). Primary antibody was omitted as staining control. Scale bar, 100 µm.

Supplementary Figure 4.

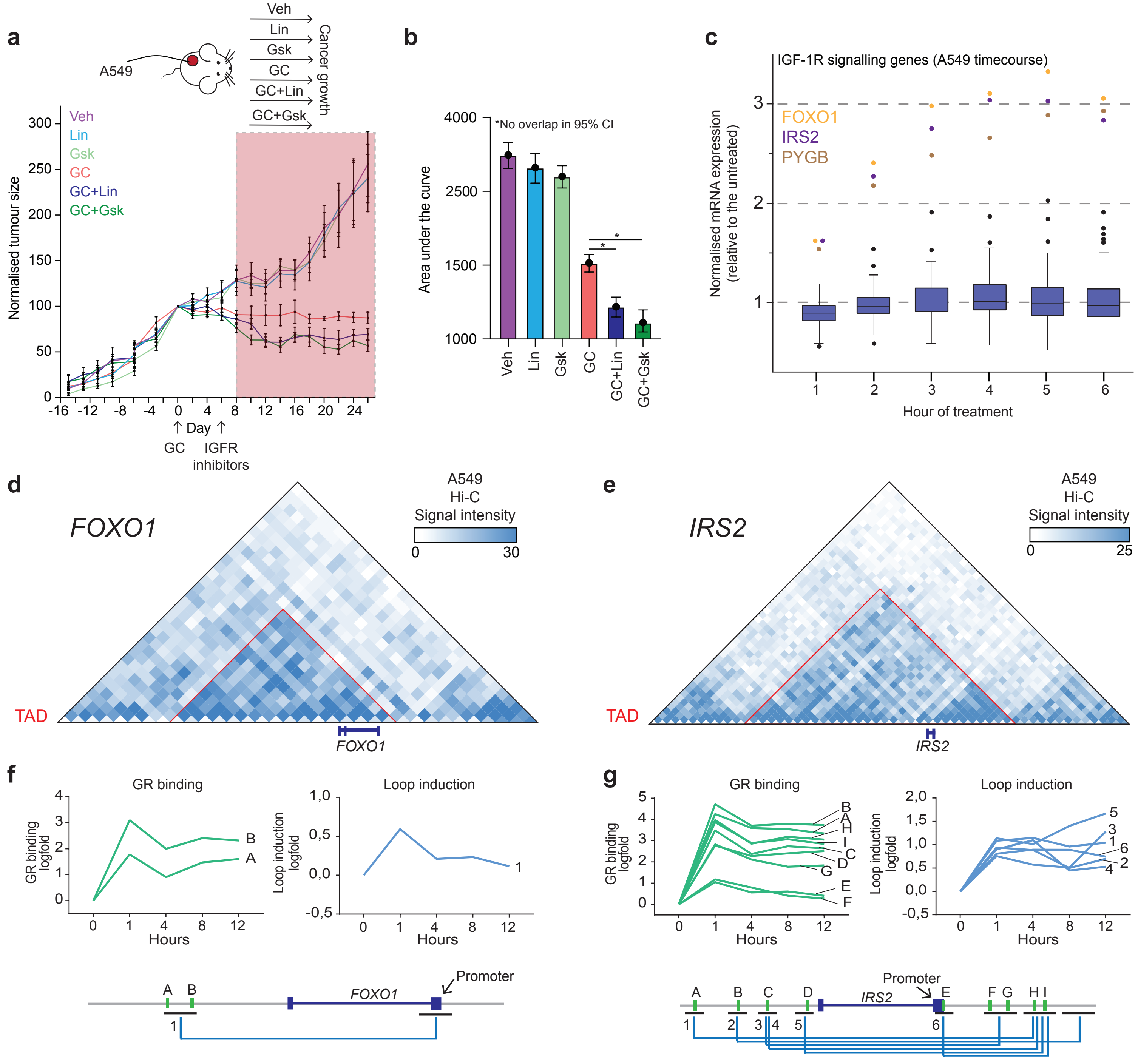

Supplementary Figure 4. Glucocorticoids increase the effectiveness of IGF-1R inhibitors *in vivo*, and regulate expression of IGF-1R signalling related genes.

a, Normalised tumour growth in xenograft models of A549 cells in NOD-SCID-γ mice treated with either vehicle (Veh=light purple), glucocorticoids (GC=red), linsitinib (Lin=blue), GSK1838705A (GSK=light green) or combination (GC+Lin=dark purple; and GC+GSK=dark green). Arrows indicate when treatment was started. Mean values ±SEM depicted (Veh n=5, Lin n=8, Dexa+Lin n=8, GSK n=5, Dexa+GSK n=7, Dexa=8). **b,** Area under curve analysis of normalised tumour size for A549 xenografts following after initiation of the secondary treatment. Area under the curve value ±SD depicted (Veh n=5, Lin n=8, Dexa+Lin n=8, GSK n=5, Dexa+GSK n=7, Dexa=8). Differences were determined based on 95% confidence interval overlaps. **c,** Normalised mRNA expression levels of *FOXO1*, *IRS2*, PYGB and other genes of the insulin signalling gene-set (M18155) throughout the time-course experiment with glucocorticoids in A549 cells (ENCSR897XFT). Box plot depicts gene expression values based on independent biological replicates (n=3). The central mark indicates the median, and the bottom and top edges of the box indicate the 25th and 75th percentiles, respectively. The maximum whisker lengths are specified as 1,5 times the interquartile range and outliers are depicted as filled circles. **d,** Hi-C contact map of A549 cell line at resolution of 40 kb of the region surrounding *FOXO1* gene (chr13:39760000-41120000). **e,** Hi-C contact map of A549 cell line at resolution of 40 kb of the region surrounding *IRS2* gene (chr13:108480000-110680000). **f-g,** Differential GR binding within loop anchors (compared to 0 hrs) across a 12hr time-course ChIP experiment in A549 cell line (top left) for the *FOXO1* (f) and *IRS2* (g) loci. Loop induction calculated from Hi-C time-course data for every loop detected in the vicinity of the FOXO1 (f) and IRS2 (g) in A549 cell line (top right). Schematic representation of the respective locus (bottom).

Supplementary Figure 5.

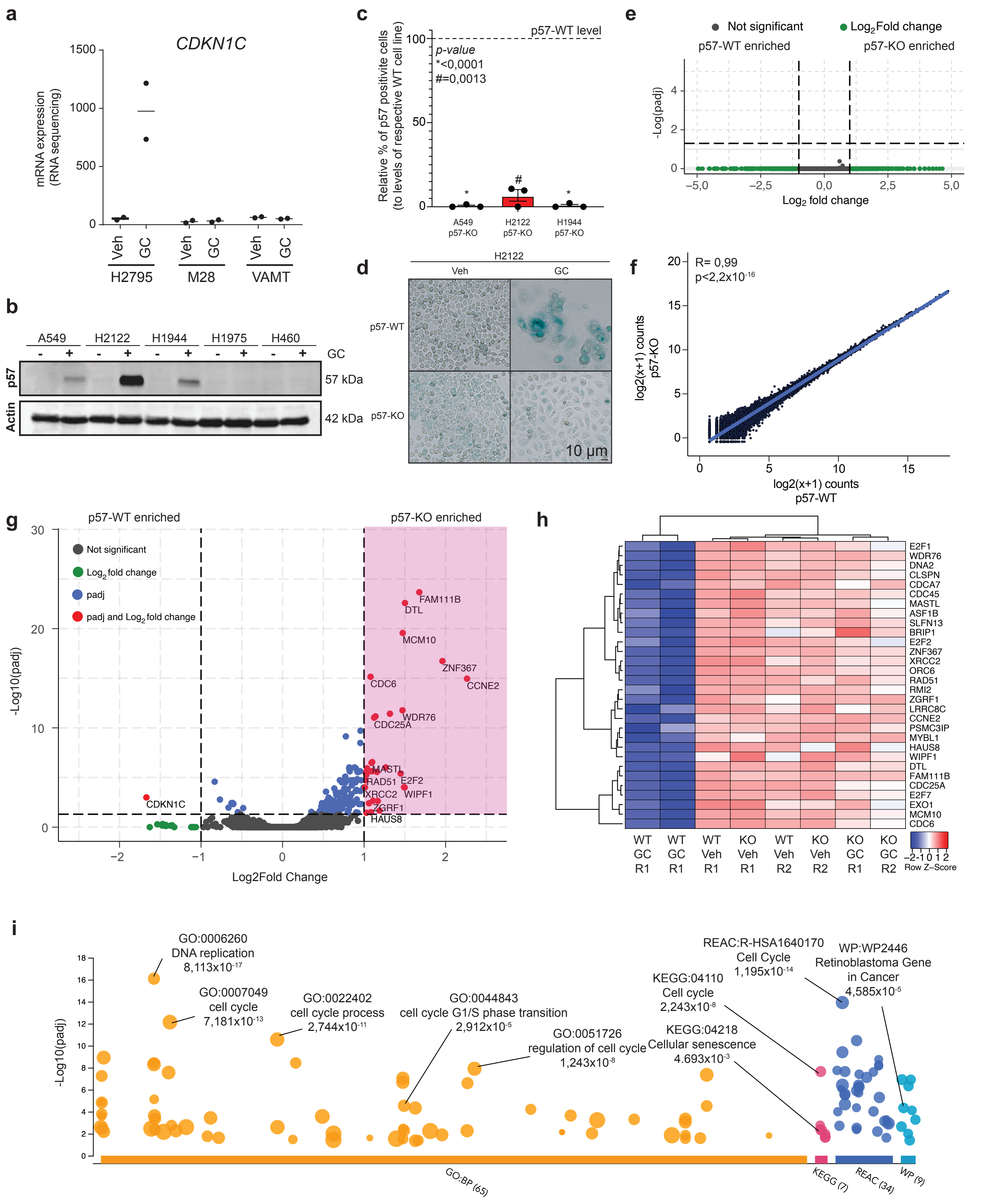

Supplementary Figure 5. Regulation of *CDKN1C* is required for glucocorticoid-induced growth arrest.

a, mRNA expression levels of *CDKN1C* in mesothelioma cell lines, untreated (Veh) or treated with glucocorticoids (GC). Mean value shown as a line (n=2). **b,** Representative western blot showing p57 expression, with actin as loading control (n=2)**. c,** Percentage of p57 positive cells in p57-KO compared to p57-WT models. Mean values ±SEM of independent biological replicates is depicted (n=3). p-values were determined by two-sided Welch's t-test. **d,** Representative images of senescence-associated β-galactosidase stained H2122 p57-WT and p57-KO cells, untreated (Veh) or glucocorticoid treated (GC) (n=2). Scale bar, 10 µm. **e,** Volcano plot depicting transcriptomic differences between vehicle-treated p57-KO and p57-WT H2122 cell lines (n=2). Adjusted p-values were determined by DESeq2 (Wald test p-values corrected for multiple testing using Benjamini and Hochberg method). **f,** Gene expression Pearson correlation of vehicle-treated p57-KO and p57-WT H2122 cell lines. Two-sided p-value is reported. **g,** Volcano plot depicting gene expression differences between GC-treated p57-WT and p57-KO models (n=2). Adjusted p-values were determined by DESeq2 (Wald test p-values corrected for multiple testing using Benjamini and Hochberg method). **h,** Heatmap depicting expression of genes enriched in GC-treated p57-KO models (labelled in red in panel **g**). R=biological replicate. **i,** Gene list analysis of all the genes enriched in GC-treated p57-KO in comparison to p57-WT cell line. p-values were determined by g:Profiler software. Source data are provided as a Source Data file.

Supplementary Figure 6.

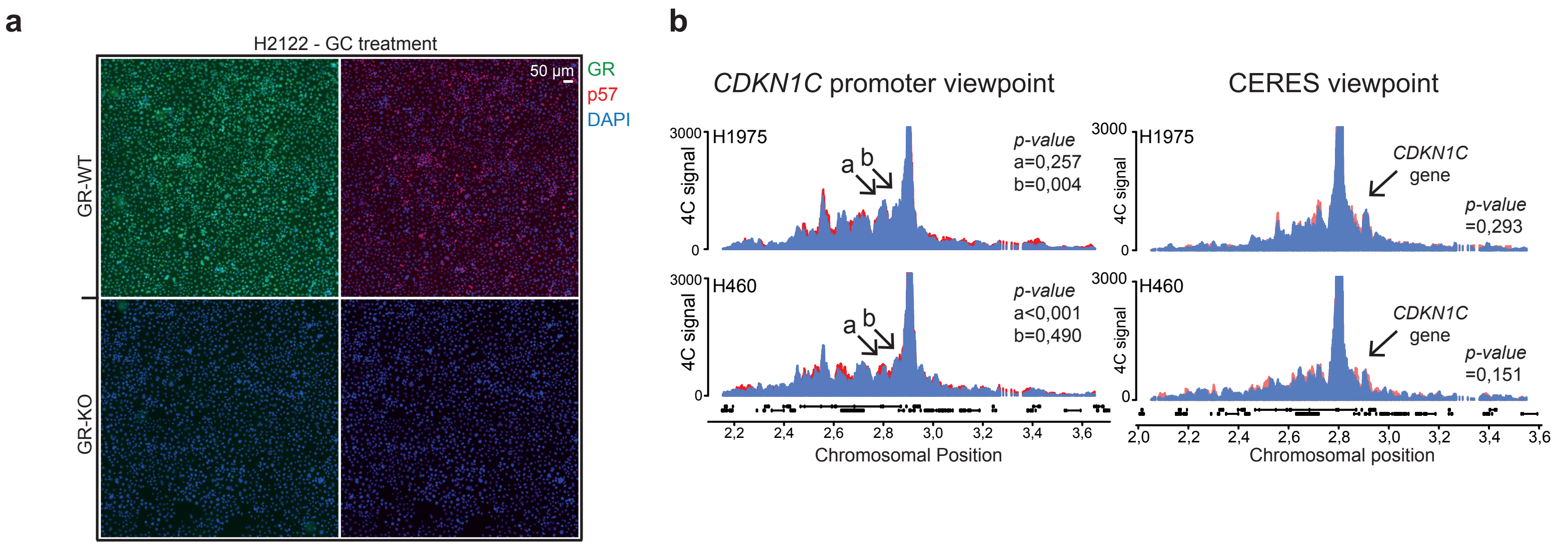

Supplementary Figure 6. Active glucocorticoid receptor is required for glucocorticoid-induced expression of *CDKN1C*.

a, Representative immunofluorescence images showing expression and localisation of GR (green) and p57 (red) in GR-WT and GR-KO H2122 cell lines, with DAPI as nuclear staining (blue) (n=2). Scale bar, 50 µm. **b,** *CDKN1C* promoter (left) and CERES (right) viewpoint 4C signal across the surrounding region (chromosome 11), under vehicle (Veh=blue) or glucocorticoid (GC=red) treatment for H1975 (top) and H460 (bottom) cell lines (n=2). p-values were determined by Wilcoxon t-test.

Supplementary Figure 7.

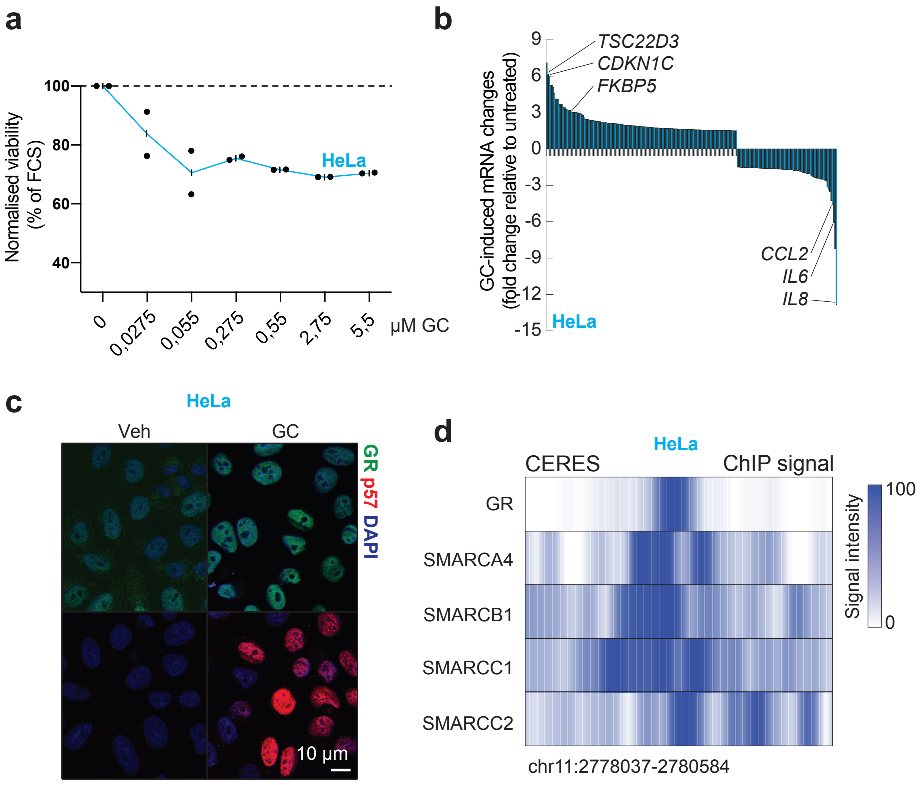

Supplementary Figure 7. Regulation of *CDKN1C* is associated with growth arrest in HeLa cells and is associated with binding of SWI/SNF complex to the CERES element.

a, Viability assay for HeLa cells treated with a range of concentrations of glucocorticoids for five days. Vertical line depicts mean value (n=2). **b,** Microarray data showing differentially expressed (-/+ 1,5-fold change) genes upon glucocorticoid treatment in HeLa cells (n=2). **c,** Representative immunofluorescence images showing expression and localisation of GR (green) and p57 (red), with DAPI as nuclear staining (blue) in HeLa cells (n=2). Scale bar, 10 µm. **d,** ChIP sequencing tracks showing binding of GR, SMARCA4, SMARCB1, SMARCC1, SMARCC2 at the CERES enhancer in HeLa cells.
